# Outcome of Childhood Cerebellar Pilocytic Astrocytoma: A Series With 20 Years of Follow Up

**DOI:** 10.7759/cureus.22258

**Published:** 2022-02-15

**Authors:** Sherif M Elwatidy, Jehad Ahmed, Minyal H Bawazir, Abdulrahman Alnasser, Jawaher Abanumy, Abdulrahman Al Shammari, Amjad Alduhaish, Safdar H Malik, Hana S Elwatidy

**Affiliations:** 1 Neurological Surgery, King Saud University, Riyadh, SAU; 2 College of Medicine, King Saud University, Riyadh, SAU; 3 Neuroscience Department, King Abdullah Medical City, Mecca, SAU; 4 Neurosurgery, King Khalid University Hospital, Riyadh, SAU; 5 Neurological Surgery, New Giza University, Cairo, EGY

**Keywords:** gross-total resection, low-grade gliomas, primary brain tumors, survival, recurrence, outcome, pilocytic astrocytoma, cerebellar, childhood, posterior fossa

## Abstract

Background: Cerebellar pilocytic astrocytoma (PCA) is one of the few CNS tumors that can be cured with gross-total removal (GTR). In this series, we had 39 patients diagnosed with cerebellar PCA, 27 patients (70%) had GTR, and mean follow-up period was 62 months with no tumor recurrence.

Objective: To assess the long-term outcome of childhood cerebellar PCA treated at our institute during the period 2000-2020 and to highlight our surgical protocol.

Methodology: Retrospective review of all patients under 18 years of age who were diagnosed with cerebellar PCA and had surgical excision between 2000 and 2020 at the Medical City of King Saud University.

Results: The study included 39 patients: 17 males and 22 females, the mean age was 8.4 years. Radiologically, the tumor was solid in eight patients, cystic in 15 patients, and mixed components were found in 16 patients. The lesion was located in the right cerebellar hemisphere in 12 patients, left cerebellar hemisphere in five patients, and midline 22 patients. The tumor size ranged from 2 to 7 cm in its greatest diameter, it was <5 cm in 13 patients and >5 cm in 26 patients. Thirty-one patients had preoperative hydrocephalus. GTR of the tumor was achieved in 27 patients and subtotal resection (STR) was done in 12 patients, 18 patients required permanent ventriculoperitoneal (V-P) shunt, and five patients had postoperative radiotherapy. Postoperative complications included infection in two patients, cerebellar mutism in two patients, and significant neurologic disability in four patients. The duration of follow-up ranged from 0 to 240 months (mean follow-up period: 62.0 months). The outcome at 10 years was good in 30 patients, fair in four patients, poor in four patients, and one patient died. Recurrence was documented in nine patients, seven of them had GTR and two had STR.

Conclusion: GTR, if achievable, is curative for childhood cerebellar PCA. Many posterior fossa surgical complications could be avoided with watertight dural closure. Although new dural substitutes are available we prefer using autologous grafts (pericranium). It is easy to harvest pericranial graft from the external ventricular drain (EVD) site. The insertion of EVD synchronously with GTR of the tumor and gradual weaning of EVD could avoid the insertion of V-P shunt.

## Introduction

Pilocytic astrocytoma (PCA) was first described by Cushing in 1931 as an independent pathologic entity [1]. About 80% of PCA are found in the posterior fossa, however, it can arise in the cerebellum, brain stem, optic tract, thalamus, and hypothalamus. It is reported that PCA accounts for 6% of all primary intracranial tumors and constitutes about 30% of all childhood posterior fossa tumors [2,3,4]. The incidence of cerebellar PCA is equal in both males and females and the mean age at presentation is 14.5 years in all patients including adults and is 6.5 years for the pediatric age group [3,4]. Cerebellar PCA can occur as an isolated tumor or be associated with other diseases such as Turcot syndrome and neurofibromatosis type 1 [5.6]. Cerebellar PCA is a slow-growing tumor, in some cases, the tumor stops growing or even regress without treatment [4,5,6]. Clinically, the classic presentation is insidious with slowly progressive symptoms over months to years with an average of three months [5,6]. The presenting symptoms are attributed to increased intracranial pressure (ICP) including headache, nausea, vomiting, papilledema, and cerebellar signs. Radiologically, it is either a solid tumor, cystic with an enhancing mural nodule, or a mixture of both [7-10]. Pathologically, it is classified as grade 1 according to the old WHO classification. In the new WHO 2021 classification, PCA is classified under the gliomas, glioneuronal tumors, and neuronal tumors, subtype circumscribed astrocytic gliomas which include PCA; high-grade astrocytoma with pyloid features; pleomorphic xanthoastrocytoma; and subependymal giant cell astrocytoma [11].

Gross-total resection (GTR) is the principal treatment, adjuvant chemotherapy and radiotherapy are not indicated after GTR in cerebellar PCA [12-14]. PCAs are known for their favorable long-term prognosis. However, unfavorable outcomes are reported to be in around 20% of cases in different large series and are attributed to brain stem invasion, surgical morbidity, and tumor recurrence [15-20].

## Materials and methods

After obtaining patient consent, and ethics committee and IRB approval, we retrospectively reviewed the medical records of all patients under 18 years of age who were diagnosed to have cerebellar PCA and had surgical excision between 2000 and 2020 at the Medical City of King Saud University. We collected demographic, clinical, radiological, operative, and follow-up data. Demographic data included age at presentation, sex, nationality, education status, and social status of the family. The clinical presentation included symptoms of raised ICP, cerebellar manifestations (gait ataxia, limb incoordination), cranial nerve palsy, seizure, and duration of symptoms. The radiological findings in computerized tomography (CT) and magnetic resonance imaging (MRI) scans pre- and postoperatively described the location of the tumor, nature of the tumor whether solid; cystic; or mixture, size of the tumor, which was classified into two groups ( <5 cm or >5 cm in maximal diameter), hydrocephalus, postoperative GTR, or residual tumor (subtotal resection (STR)). Operative findings include insertion of an external ventricular drain (EVD), the extent of vermian incision, tumor vascularity and consistency, brain stem infiltration, opening the aqueduct of Sylvius, establishing a free CSF pathway, and achieving watertight dural closure. Postoperative complications as well as histopathological diagnosis and follow-up data were all collected in an Excel datasheet. Long-term outcomes according to Glasgow outcome score (GOS) and Karnofsky score including tumor recurrence, neurological deficits, cognitive complications, and quality of life, including school performance, were reviewed and documented.

## Results

The study included 39 patients: 17 males (43.6%) and 22 females (56.4%), the mean age was 8.4 years and the median age at the time of diagnosis was 8 years. Patients presented with headache (95%), nausea and vomiting (90%). Papilledema was present in 60% of patients, and 36.6% of patients had diplopia. Cerebellar signs were present in 21 patients (53.8%) and three (7.7%) patients presented with generalized seizures.

**Figure 1:**
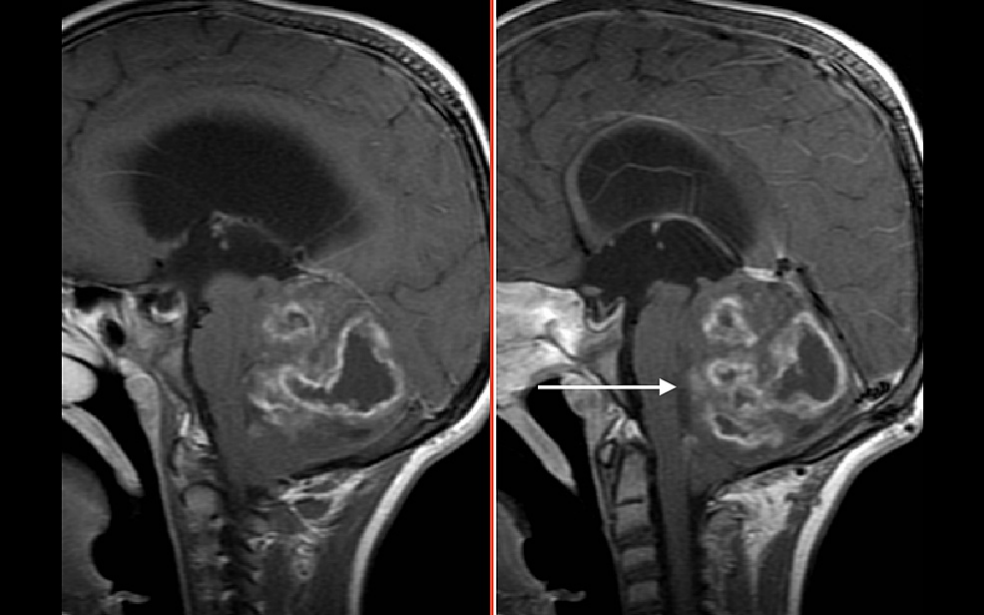
MRI of the brain Sagittal T1 image with contrast showing the tumor compressing the fourth ventricle (white arrow) and the brain stem

**Figure 2:**
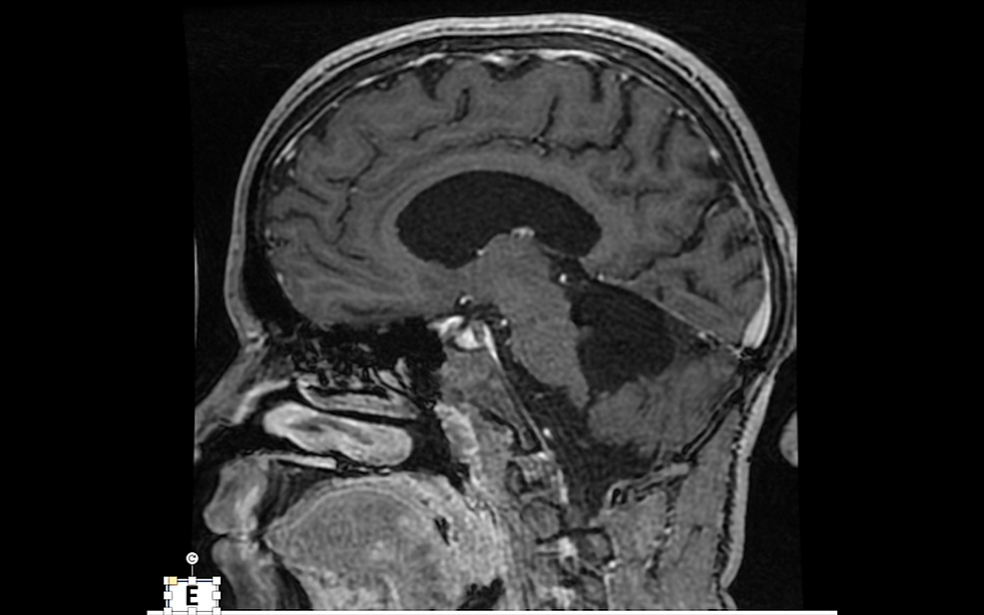
MRI scan of the brain Sagittal T1 with contrast taken 2 years after surgery showing no residual or recurrent tumor

Radiologically, all patients had MRI before surgery, the tumor appeared solid in eight (20.5%) patients, cystic in 15 patients (38.5%), and a mix of solid and cystic components in 16 (41%) patients. The lesion was located in the right cerebellar hemisphere in 12 (30.8%) patients, left cerebellar hemisphere in five (12.8%), and midline in 22 (56.4%) patients. Tumor calcification was seen in CT scans of two patients. The tumor size ranged from 2 to 7 cm in its greatest diameter, it was less than 5 cm in 13 (33.3%) patients and more than 5 cm in 26 (66.7%) patients. Thirty-one (79.5%) patients had preoperative hydrocephalus. GTR of the tumor was achieved in 27 (69.2%) documented by postoperative MRI scans (Figures 1-2) and STR was achieved in 12 (30.8%) patients. 

Eighteen patients (46.2%) required permanent ventriculoperitoneal (V-P) shunt; nine of them had GTR of the tumor and the other nine had STR. Postoperatively, five patients (12.8%) had radiotherapy and no one had received chemotherapy. Postoperative complications included infection in two patients (5.1%), cerebellar mutism in two patients (5.1%), significant neurologic disability in four patients (10.2%), and subdural collection (treated conservatively) in four (10.2%) patients. The duration of follow-up ranged from 0 to 240 months (mean follow-up period was 62.0 months). The outcome at 10 years was good in 30 (76.9%) patients, fair in four (10.3%) patients, poor in four (10.3%) patients, and one patient had (2.6%) died. Recurrence was documented in nine patients (23%), seven of them had GTR and two had STR. The outcome was good in 50% of patients with recurrent tumors and 90% of patients with no recurrence. Table 1 summarizes the outcome of patients in relation to clinical, radiologic, and surgical findings.

**Table 1:** The outcome of patients in relation to clinical, radiologic, and surgical findings The total good outcome for each variable was 30 and total poor outcome for each variable was 9. V-P shunt: ventriculoperitoneal shunt

Variable	Good outcome	Poor outcome
Sex		
Male	12	5
Female	18	4
Radiology type		
Solid	6	2
Cystic	10	5
Mixed	14	2
Radiology (site of the lesion)		
Right hemisphere	11	1
Midline	15	7
Left hemisphere	4	1
Radiology (tumor size)		
< 5 cm	7	6
> 5 cm	23	3
Total	30	9
Amount of resection		
Gross-total removal (GTR)	24	3
Subtotal resection (STR)	6	6
Hydrocephalus		
No V-P shunt	18	3
V-P shunt	12	6
Recurrence		
No recurrence	24	6
Recurrence	6	3

## Discussion

Cerebellar PCA is one of the few CNS tumors that can be cured with GTR. In this series, we had 39 patients diagnosed with cerebellar PCA, 27 patients (70%) had GTR and were followed up for an average of 62 months with no tumor recurrence.

Pathologically, PCA is classified as grade 1 according to the old WHO classification, which in the new WHO 2021 classification is put under the gliomas, glioneuronal and neuronal tumors, subtype circumscribed astrocytic gliomas which include PCA; high-grade astrocytoma with pyloid features; pleomorphic xanthoastrocytoma; and subependymal giant cell astrocytoma. There is a common agreement in the literature that the best outcome is achieved with total surgical resection with 10-year survival rates exceeding 90% [12,21,22]. Keeping this target in mind, we adopted a policy for management of cerebellar PCA that entails maximal safe resection of tumor (GTR) unless there is brain stem invasion which could be anticipated both clinically and radiologically. Clinically, brain stem invasion is suspected in presence of cranial nerve palsy (predominantly 6th and 7th nerve palsy) and radiologically by the presence of a high signal in the brain stem detected on T2 and fluid-attenuated inversion recovery (FLAIR) series [7,8,23-25].

Our protocol for the management of a patient presenting with acute hydrocephalus is to book the patient for urgent insertion of EVD and posterior fossa craniotomy for tumor excision in the same session. After positioning the patient on the operating table (Park bench position with the head rotated so the face looks toward the floor), we insert the EVD in the right occipital horn through a separate incision centered over Keen’s point. Before making the burr hole, we harvest a pericranial graft which we use for closure of the posterior fossa dura. After surgery, we keep the EVD open to drain CSF at a rate of 10 ml/hour for 5-7 days. Gradually, we wean the EVD by the sequential rise of its height above the head and monitoring the amount of daily CSF drainage and we clamp the drain for 48 hours before its removal. The drain is removed after clamping for 48 hours if the patient did not develop manifestations of raised ICP, CSF collection/leak at the wound and CT scan does not show active hydrocephalus. V-P shunt is inserted if the patient did not tolerate the process of weaning the EVD or if he developed active hydrocephalus after its removal. With this protocol, 54% of patients in our series did not require permanent CSF diversion (V-P shunt). To reduce the risk of persistent hydrocephalus, we adequately suck all the blood in the cisterna magna, subarachnoid space, and 4th ventricle, and use plenty of saline wash to clear all blood clots and minimize the use of hemostatic materials left in the 4th ventricle, together with watertight dural closure.

From previous reports and in our experience, many postoperative complications following posterior fossa surgery are attributed to the inability to achieve watertight dural closure leading to CSF collection and leak from the wound with its sequelae. To avoid this complication, we harvest a pericranial graft during insertion of the EVD and use it for dural closure under a microscope using 5/0 nonabsorbable monofilament suture and enforce it with a sealant. Thirty-one (79.5%) patients had preoperative hydrocephalus, 18 of them (36.2%) required permanent V-P-shunt, while 13 had temporary EVD and did not require a shunt.

Most neurosurgeons would agree that solid tumors are easier to resect than cystic ones because it is easier to create a pseudo cleavage plane around solid tumors which enables total resection. We achieved GTR in 69.2% of cases. Recently (during the last 5 years), intraoperative MRI was introduced in our operating room and it was of great help in achieving GTR of tumors. In our series, we achieved GTR in 27 (69.2%) and STR in 12 (30.8%) patients which were documented by MRI scans. Tumor recurrence was documented in nine patients (23%), four of them had GTR and five had STR. Two-thirds of patients with recurrent tumors had a good outcome.

Tumor recurrence in cerebellar PCA ranges from 0 to 33% in different series with most recurrences seen within 4-5 years of the primary surgery and with lower incidence of recurrence (6-9%) after GTR [23-27]. In our series, nine patients (23%) had tumor recurrence, seven of them had GTR and two had STR. The outcome was good in 50% of patients with recurrent tumors and 90% in patients with no recurrence. The poor outcome in our series (23%) is attributed to brain stem invasion, persistent hydrocephalus, subtotal tumor resection, and tumor recurrence.

Limitations of the study are the relatively small number of cases, and in few patients, a short period of follow-up due to logistic issues.

## Conclusions

GTR, if achievable, is a curative for childhood cerebellar PCA. Most posterior fossa surgical complications could be avoided with watertight dural closure. Although new dural substitutes are available, we prefer using autologous grafts (pericranium). It is easy to harvest from the EVD site and heals quickly with a lower risk of CSF leak. The insertion of EVD synchronously with tumor excision and gradual weaning of EVD after GTR of tumor could avoid permanent V-P shunt.
